# Geospatial heterogeneity of hotspots for incidence and late-stage diagnosis of breast, colorectal, and lung cancer

**DOI:** 10.1007/s10552-026-02170-z

**Published:** 2026-05-26

**Authors:** Andrew P. Loehrer, Heather A. Carlos, Julia E. Weiss, Qianfei Wang, Chelsea V. Leversedge, Julia E. Katter, Joseph D. Phillips, Dana Ferrari-Light, Tracy Onega, Xun Shi

**Affiliations:** 1 Dartmouth-Hitchcock Medical Center, Lebanon, NH USA; 2 Geisel School of Medicine at Dartmouth, Hanover, NH USA; 3 Dartmouth Cancer Center, Lebanon, NH USA; 4 The Dartmouth Institute for Health Policy and Clinical Practice, Lebanon, NH USA; 5 University of Utah, Huntsman Cancer Institute, Salt Lake City, UT USA; 6 Department of Geography, Dartmouth College, Hanover, NH USA; 7 Dartmouth-Hitchcock Medical Center, One Medical Center Drive, Lebanon, NH 03756 USA

**Keywords:** Hotspot, Cancer incidence, Access to care, Cancer control, Prevention

## Abstract

**Purpose:**

Cancer control relies on the identification of populations at risk (hotspots) of new or late-stage cancer diagnoses. However, the extent to which hotspots differ between cancer sites or between outcome measures has been poorly characterized. We sought to determine the geospatial heterogeneity of hotspots of breast, colorectal, and lung cancer incidence and late-stage diagnoses.

**Methods:**

We identified adult patients diagnosed with female breast, colorectal, and lung cancer between 2010 and 2019 in Indiana. To identify hotspots for incidence and late-stage diagnoses, we disaggregated the patient residential location information from the Census block group level to the approximated individual point level. Statistically significant hotspots were identified with kernel ratio estimation. The total areas of hotspots and the overlap between hotspots were compared.

**Results:**

133,773 patients diagnosed with breast (n = 54,903), colorectal (n = 28,594), and lung (n = 50,276) cancer were included. Geospatial visualization demonstrated marked spatial deviation, with little overlapping area between incidence and late-stage hotspots for all three cancer sites (1–266 km^2^). However, there was slightly greater overlap in late-stage hotspots between the different cancer sites, with total overlapping hotspot areas ranging from 24 to 516 km^2^.

**Conclusions:**

Our results demonstrate considerable geospatial heterogeneity of hotspots between different outcome measures and different sites of cancer. The use of disaggregated spatial data enables a more granular, precise comparison of cancer hotspots. The greatest overlap was seen between incidence hotspots of breast and colorectal cancer, which suggests similar spatial drivers and the potential for coordinated cancer control strategies.

**Supplementary Information:**

The online version contains supplementary material available at 10.1007/s10552-026-02170-z.

## Introduction

Cancer prevention and control efforts are contingent on the ability to identify populations and communities at greatest risk of cancer development and delayed, late-stage disease at the time of diagnosis [1–3]. The utilization of aspatial data has repeatedly demonstrated specific population attributes associated with adverse cancer outcomes, including lower socioeconomic status, higher concentration or segregation of minority communities, and residence in more rural areas of the country [4–6]. While constructive in understanding general risks of adverse outcomes, these demographic characteristics alone fail to differentiate which specific communities are associated with such outcomes [7, 8]. Geospatial visualization, which incorporates spatial data, is critical in identifying specific communities experiencing worse than expected outcomes compared to surrounding areas.

Prior work has demonstrated areal clusters of hotspots for breast, colorectal, and lung cancer diagnoses and presentation with late-stage disease [1, 9, 10]. Additionally, many federal, state, and other professional cancer programs regularly evaluate cancer outcomes using various geospatial methodologies with larger areal units of evaluation, such as ZIP codes or counties [11–13]. However, significantly less is known about how to identify smaller areas of specific risk that influence both the incidence of cancer and access to timely diagnosis. Furthermore, knowledge gaps remain regarding the similarity and overlap of hotspots between different cancer outcome measures and different cancer types. Consequently, policy or care delivery changes aimed at cancer prevention and control may fail to locate the appropriate communities to target or outcome measures to evaluate select interventions. Alternative approaches, such as using underlying population density to disaggregate spatial data, have been proposed for more precise and stable estimates of disease hotspots [14–16]. However, their ability to discriminate between hot spots of different outcome measures or between different cancer sites remains unclear.

The primary objective of this study is to evaluate the geospatial heterogeneity of hotspots of incidence and late-stage diagnoses of breast, colorectal, and lung cancer. Factors associated with higher incidence do not necessarily align with risk factors for presenting with later-stage cancer at the time of diagnosis, especially for breast cancer. Concurrently, the local factors contributing to hotspots of later-stage cancer may not overlap for different cancer sites. We hypothesize that hotspots will vary between sites of cancer (breast, colorectal, or lung) as well as outcome measures (incidence and late-stage diagnosis). Given more closely related drivers of timely access to care, we hypothesize that there will be greater overlapping areas of late-stage hotspots between the different cancer sites.

## Materials & methods

### Cohort development

**Fig. 1:**
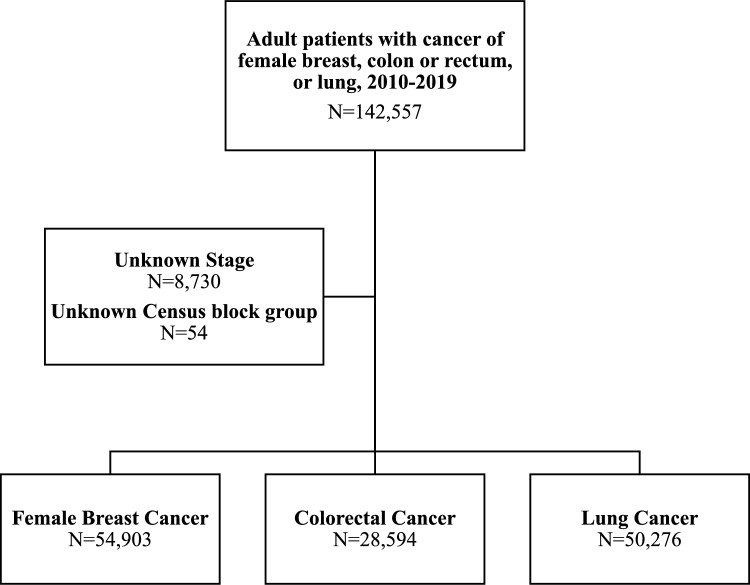
Cohort development from the Indiana State Cancer Registry (ISCR), STROBE Chart

Using data from the Indiana State Cancer Registry (ISCR), we identified all female patients 18 years or older diagnosed with breast (female only), colorectal, and lung cancer from 1/1/2010 through 12/31/2019 (Fig. 1). The ISCR captures nearly 100% of all new cancer diagnoses each year and is maintained in accordance with national standards set by the North American Association of Central Cancer Registries. The International Classification of Disease-oncology site and pathology codes were used to identify the cancer cases. Patients were excluded if they had missing residential location at the level of the United States Census block group. This study was deemed exempt from review by the Dartmouth-Hitchcock Institutional Review Board.

### Primary outcomes

Our primary outcomes were the incidence of breast, colorectal, or lung cancer and presentation with late-stage cancer at the time of diagnosis. Late-stage cancer diagnosis was determined for each cancer site using the American Joint Committee on Cancer (AJCC) 7th and 8th Edition staging system variables as captured in the ISCR [17]. A hierarchical calculation using both ISCR pathology and clinical staging data within the two time periods 2010–2017 and 2018–2019 defined the stage [18]. This staging algorithm used pathology staging first and if pathology was absent, then clinical staging. An indicator variable for late-stage cancer diagnosis differentiated individuals classified as stage III or IV from those classified as stage 0, I, or II.

### Patient characteristics

Patient demographic and clinical characteristics were used to describe our cohort using the statistical software SAS 9.4 package by cancer type [19]. ISCR’s variables included patients’ age, sex, race/ethnicity, comorbidities, primary payer, 2010 census tract and block group, residential rural–urban community area (RUCA). A combined race/ethnicity variable was created from the ISCR’s variables with the following categories: Non-Hispanic White, Non-Hispanic Black, Non-Hispanic Other/Unknown and Hispanic (any race). An Elixhauser comorbidity index software tool package utilizing the ISCR’s comorbidity and secondary diagnoses registry data produced a comorbidity index, and grouped into three categories (0, 1 or 2 or more) for the number of comorbidities [20]. Patients’ primary-payer insurance classifications were collapsed into six categories: (1) private insurance, (2) Medicaid, (3) Medicare, (4) uninsured or self-pay, (5) other insurance, and (6) unknown. The RUCA was established at the level of the patients’ 2010 census tract, creating four categories: metropolitan, micropolitan, small town, and rural areas [21].

### Geospatial visualization and hotspot determination

The analytical procedure adopted in this study for hotspot determination is described briefly here, with greater detail in previous publications [14, 22, 23]. Geospatial visualization and hotspot were performed using ArcGIS Pro and ArcHealth, a software package developed as an extension of ArcGIS Pro [14]. The essential method we used in this study for quantifying the local disease intensity is called kernel ratio estimation (KRE) [14, 22]. The basic idea of KRE is to define a circular neighborhood (kernel) around a disease case location and compare the actual patients-to-population ratio with simulated (expected) ratios [14, 22, 23].

For the background population, we used LandScan™ USA, a gridded population dataset available through the Oak Ridge National Laboratory [24]. Since the LandScan data only has the population count for each raster cell, we applied the age information from the US Census block group to the LandScan™ data by assuming the population in a raster cell has the same age structure as the corresponding Census block group. Both the modified LandScan data and the patient cohort were stratified into three age groups (Online Resource 1) to mitigate the effects of confounding by age. The age groups reflect the United States Preventive Services Task Force cancer screening guidelines in place at the time of diagnosis [25].

The analytical procedure is briefly described here, and further details can be found in previous publications, and a schematic of the procedure is available in Online Resource 2 [14, 21, 22]. The kernel method requires point locations, but the cohort data from the ISCR were aggregated at the block group level. To take advantage of KRE, we disaggregated the block group-level patient counts to points using a restricted (to the associated block group) and controlled (by the background (LandScan) population) Monte Carlo (RCMC) process and then ran KRE with a 250-person adaptive bandwidth on the disaggregated points [26]. The 250-person adaptive bandwidth provided a geographically granular areal measurement while incorporating enough people for stability of estimates. Compared to other bandwidth options, the 250-person adaptive bandwidth best balanced discernability of spatial variation of outcomes and the statistical stability of estimates (incorporating enough patients in the kernel). Since this disaggregation is a randomizing process, to estimate the uncertainty in this process, we ran RCMC 10 times, considering the output of each to represent the actual locations of the patients.

To estimate the statistical significance of the local disease rate calculated by KRE, we generated simulated (expected) patient locations through an unrestricted (not limited to the block group polygon) but controlled (by the statewide background population) Monte Carlo process (UCMC) and then ran the KRE on the simulated patient locations using the same adaptive bandwidth applied to the patient locations disaggregated by RCMC. The KRE process was run on each of the three age groups, and then the resulting estimates from the age groups were integrated into an overall estimate following the principle of direct adjustment. The statistical signifi cance of the result of each RCMC iteration was determined (i.e., the actual patient-to-population ratio at each location) and result was compared to 199 results from the UCMC simulations to determine the overall p-value for each location. A location (represented by a raster cell) is considered to be within a hotspot area if the mean of its 10 p-values plus 2 standard deviations was < 0.01. Due to the prohibitively large size of output data for each hotspot, ArcHealth does not provide confidence intervals for each hotspot and as such none are reported in the results.

To further characterize the hotspots, we compared the absolute size and distribution of the identified hotspots both by cancer site and outcome measure. The sizes of the hotspot areas were compared to the total area of the state, and the area of overlap between outcome measures for each cancer site as well as for the same outcome measure for different cancer sites was also calculated. The areas were reported in absolute square kilometers and as a percentage of the total area of the state. We also attributed case and late-stage diagnosis case counts to the hotspot areas by apportioning the block group’s counts based on the population distribution. From this, we established rates of late-stage diagnosis in hotspot and non-hotspot locations. A continuous hotspot area (which may consist of multiple hotspot pixels) with fewer than 11 cases was suppressed for patient confidentiality and stability purposes [27].

## Results

Our final analytic cohort included 133,773 patients diagnosed with breast (n = 54,903), colorectal (n = 28,594), and lung (n = 50,276) cancer in Indiana between 2010 and 2019 (Table 1). Patients with breast cancer had a younger average age at diagnosis (62.3 years), a higher proportion of non-Hispanic Black patients, fewer comorbidities, and a higher proportion of patients with private insurance coverage. Conversely, patients with lung cancer were more likely to be non-Hispanic White, have one or more comorbidities, and have Medicare insurance coverage.

**Table 1:** Indiana cohort demographic and clinical characteristics among those with breast, colorectal and lung cancer (N = 142,557) and diagnosed between 1/1/2010 and 12/31/2019

	Breast (N = 54,903)	Colorectal (N = 28,594)	Lung (N = 50,276)
Age mean (std)	62.3 (13.1)	67.0 (13.4)	68.7 (10.7)
Female	54,903 (100)	13,441 (47.0)	22,978 (45.7)
Race/ethnicity			
Non-hispanic white	48,433 (88.3)	25,503 (89.2)	46,088 (91.7)
Non-hispanic black	4,600 (8.4)	2,185 (7.6)	3,519 (7.0)
Non-hispanic other/unknown	862 (1.6)	434 (1.5)	329 (0.6)
Hispanic (any race)	1,008 (1.8)	472 (1.7)	340 (0.7)
Number of comorbidities			
0	19,894 (36.2)	9,977 (34.9)	15,546 (30.9)
1	6,375 (11.6)	5,589 (19.5)	11,491 (22.9)
2 +	2,547 (4.6)	3,598 (12.6)	8,348 (16.6)
Unknown	26,087 (47.5)	9,430 (33.0)	14,891 (29.6)
Insurance coverage			
Private	19,990 (36.4)	7,009 (24.5)	8,078 (16.1)
Medicaid	2,860 (5.2)	1,364 (4.8)	3,115 (6.2)
Medicare	23,931 (43.6)	15,730 (55.0)	32,005 (63.7)
Uninsured/self-Pay	1,016 (1.9)	743 (2.6)	1,386 (2.8)
Other	5,822 (10.6)	2,810 (9.8)	4,259 (8.4)
Unknown	1,284 (2.3)	938 (3.3)	1,433 (2.8)
Rural urban commuting area			
Metropolitan	42,889 (78.1)	21,017 (73.5)	37,224 (74.0)
Micropolitan	7,300 (13.3)	4,596 (16.0)	8,093 (16.1)
Small Town	3,087 (5.6)	1,958 (6.9)	3,303 (6.6)
Rural	1,627 (3.0)	1,023 (3.6)	1,656 (3.3)
Stage at diagnosis			
0	9478 (17.3)	1,877 (6.6)	87 (0.2)
1	25,130 (45.8)	6,394 (22.3)	10,807 (21.5)
2	13,124 (23.9)	6,737 (23.6)	4,351 (8.7)
3	4,348 (7.9)	7,461 (26.1)	10,973 (21.8)
4	2,823 (5.1)	6,125 (21.4)	24,058 (47.9)
Unknown, occult			
Late-stage diagnosis			
No	47,732 (86.9)	15,008 (52.5)	15.245 (30.3)
Yes	7,171 (13.1)	13,586 (47.5)	35,031 (69.7)

**Fig. 2:**
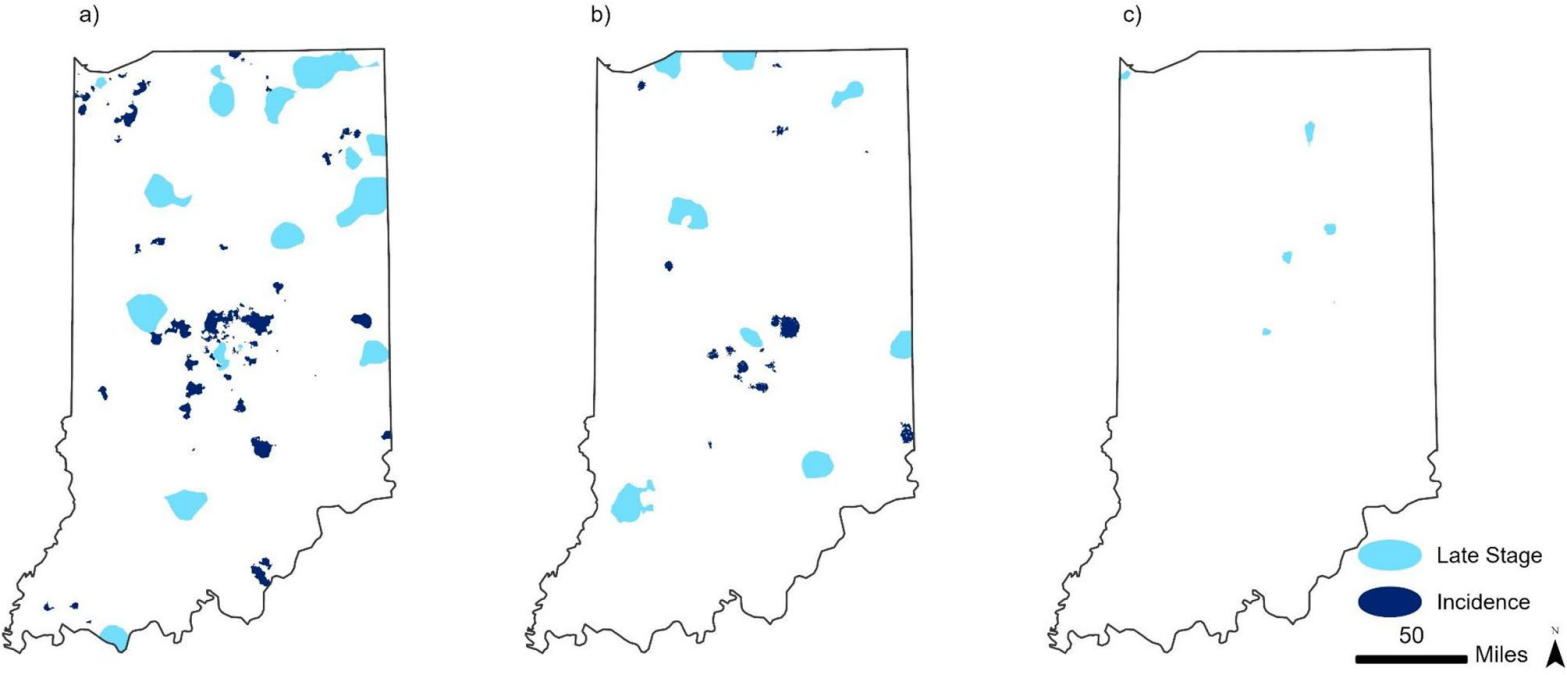
Geospatial hotspots of incidence and late-stage cancer in Indiana for **a** breast, **b** colorectal, and **c** lung cancer, 2010–2019

Overall, we identified 72, 39, 37 incidence hotspots and 15, 8, and 5 late-stage hotspots for breast, colorectal, and lung cancer, respectively (Fig. 2). The average proportion of late-stage breast cancer in these hotspots was 17.6% (range of 10.4%–25.5%) compared to 12.8% in non-hotspot areas of the states. For patients with colorectal cancer, the average proportion of late-stage cancer in hotspots was 51.0% (range of 47.1%–57.6%) compared to 47.2% in non-hotspot areas of the states. In lung cancer, the average proportion of late-stage disease in these hotspots was 66.6% (range of 56.7%–74.1%) compared to 69.7% in non-hotspot areas of the states.

The hotspots for breast cancer incidence totaled approximately 2892 km^2^ and tended to be on the outskirts of major cities, including Indianapolis, Fort Wayne, Gary, and South Bend, as well as in major college towns of Bloomington and Lafayette (Fig. 2a and Table 2). Conversely, there was a larger total area of hotspots (5473 km2) of late-stage diagnoses, and these tended to be closer to or within cities, including Indianapolis, South Bend, and Gary. Overlap with late-stage hotspots occurred in an area of only 22 km^2^ (Table 2).

**Table 2:** Area for individual hotspots and the overlap of hotspots within and between cancer sites

	Primary hotspots (square kilometers)			Overlap of hotspots between cancer sites (square kilometers)			
	Breast	Colorectal	Lung	Breast—colorectal	Breast-lung	Colorectal-lung	Breast-colorectal-lung
Incidence hotspots	**2892**	**2377**	**531**	516	48	36	12
Late-stage hotspots	**5473**	**2611**	**248**	278	59	24	0
Overlap of hotspots (square kilometers)	22	266	1				

Incidence hotspots for colorectal cancer encompassed 2377 km^2^ (Table 2). In evaluating hotspots for colorectal cancer, there appears to be closer proximity and more overlap between incidence and late-stage hotspots (Fig. 2b). Incidence and late-stage colon cancer hotspots occur both within and outside of metropolitan areas. Overlap between incidence and late-stage hotspots occurred in a total area of 266km^2^ (Table 2).

For lung cancer, we found fewer and smaller hotspots for both incidence and late-stage lung cancer (Fig. 2c). Furthermore, the area of the incidence hotspots for lung cancer totaled 531 km^2^. Similarly, there was a modest total area of hotspots for late-stage lung cancer encompassing a total area of 248 km^2^ which was largely located in more rural areas plus the East Chicago and Hamond in Northwest Indiana (Fig. 2c and Table 2).

**Fig. 3:**
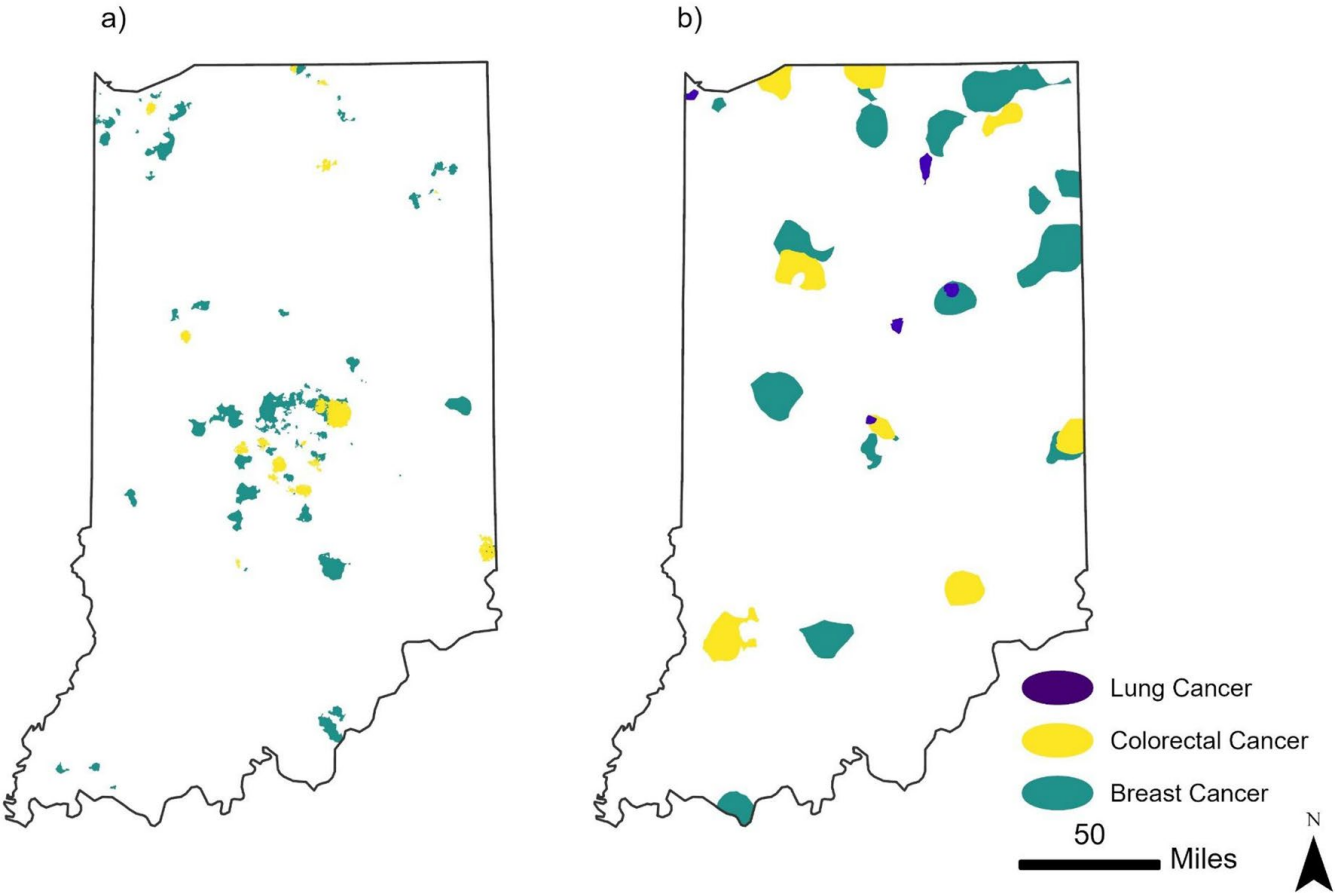
Geospatial Hotspots of **a** incidence, **b** late-stage diagnosis with breast, colorectal, and lung in Indiana, 2010–2019

The relationships of incidence and late-stage hotspots between the different cancer sites are presented in Fig. 3. The incidence hotspots for breast and colorectal cancer had the highest degree and area of overlap (516 km^2^ of total overlapping area) and were in closer proximity to each other, especially in the center portion of the state (Fig. 3a and Table 2). There appear to be more isolated hotspots of colorectal and breast cancer scattered in the more rural southern or northeast portion of the state. Shifting to comparing late-stage hotspots for the different cancer sites, late-stage breast and colorectal cancer hotspots had the greatest area of overlap (encompassing 278 km^2^) followed by modest overlap between breast and lung as well as between colorectal and lung (59 km^2^ and 24 km^2^, respectively; Fig. 3b and Table 2). There was no area in the state that had overlap of late-stage hotspots for all three cancer sites.

## Discussion

In this study of 133,773 adult patients diagnosed with breast, colorectal, or lung cancer between 2010 and 2019 in Indiana, we demonstrated considerable heterogeneity in hotspots both between different outcome measures as well as between different cancer types. Within cancer sites, colorectal cancer had the greatest degree of overlapping incidence and late-stage hotspots, but with a small area of 266 km^2^. Comparing hotspots between cancer sites, the overlap for incidence hotspots was greatest for breast and colorectal cancer hotspots (516 km^2^) and less between lung and either breast or colorectal cancer hotspots (48 km^2^ and 36 km^2^, respectively). The greatest degree in overlap between late-stage hotspots was between breast and colorectal cancer hotspots, with an area of 278 km^2^. Collectively, these results demonstrate marked variation both within and between cancer sites in localizing hotspots for two outcome measures along the cancer continuum. Given Indiana’s total estimated area of approximately 94,000 km^2^, each of these areas of overlap represents well below 1% of total state area. Further, the Monte Carlo simulation employed in hotspot determination does have a degree of uncertainty such that areas smaller than 1% of total state area may be expected to occur by chance. Thus, such small areas of geographic overlap reinforce the primary findings of heterogeneity of hotspots between outcome measures for the same cancer site and between the same outcome measures for different cancer sites.

Cancer control and prevention efforts rely on appropriate identification of populations at risk of developing or failing to receive treatment for cancer [1, 8, 28]. Traditional polygon-based geospatial methodology assumes uniform distribution of outcomes across large areas with considerable variation in population demographics, environmental exposures, and health care services [28–30]. Our use of disaggregated data and raster-based methodology demonstrated considerable geospatial heterogeneity of hotspots across the state of Indiana. These results reinforce the urgency to use place-based consideration in understanding drivers of and variation in cancer inequity across the continuum of disease [31].

The heterogeneity of hotspots seen in this study is most likely due to marked variation in the drivers of cancer development and cancer care delivery in general and in different cancer sites [32, 33]. Breast cancer incidence hotspots were located largely on the outskirts or edges of cities and in communities with the lowest degree of socioeconomic deprivation. This is consistent with prior work showing the inverse relationship between poverty and the incidence of breast and colorectal cancer [34]. Although within a relatively small area, there was more overlap between breast and colorectal incidence hotspots as compared to either site overlapping with lung cancer incidence hotspots. Conversely, lung cancer incidence is known to have more of a direct correlation with poverty [35]. As compared to breast or colorectal cancer, the vast majority of lung cancers are attributable to the patient-level behavior of smoking [36]. Our results demonstrated very few and a small area of lung cancer incidence hotspots in Indiana. Over this study period, Indiana was in the top third of states in terms of adult smoking rates and in the top 15% of states in terms of lung cancer incidence [37, 38]. As such, there may be more ubiquitous smoking rates across the state leading to lower probability of one area having disproportionately greater lung cancer risk than others (“hotspots”).

Compared to incidence hotspots, there was a greater area of late-stage hotspots for breast and colorectal cancer, but contrary to our hypothesis there was little overlap between late-stage hotspots between different cancer sites. Prior research demonstrated shared drivers of late-stage diagnosis, especially for breast and colorectal cancer which have greater availability and accessibility of screening services [4, 10, 13]. Geographic and socioeconomic variation in access to care, including screening services, has been well documented for all breast, colorectal, and lung cancer [4–6, 10, 12, 13, 28, 35, 36]. Indiana, similar to the rest of the country, has a relatively low lung cancer screening rate of approximately 17% of high-risk patients [39]. Thus, we are not necessarily surprised to see few hotspots of late-stage lung cancer diagnosis and little difference in rates of late-stage disease between hotspots and non-hotspots given more uniform (if not discouraging) rates of late-stage diagnosis across the state. Of note, however, is that the largest area of overlapping late-stage hotspots was between breast and colorectal cancer, suggesting a potential opportunity to coordinate screening services in these higher-risk patients and areas of the state [40]. Importantly, these areas of overlapping hotspots, especially for incidence hotspots, may not necessarily imply shared biological or even environmental etiologies, but may rather reflect structural inequities in terms of access to care across the cancer care continuum. However, future cancer control efforts should seek to identify overlapping areas as a means to create more efficient and effective interventions to prevent cancer development or improve timely diagnosis, using traditional screening modalities, newer multicancer detection tests, or other modalities [40–44]. Specifically, community-engaged outreach and targeted interventions to improve cancer screening use geospatial hotspot mapping of cancer incidence to identify where the most impact can be achieved [45, 46]

These results have several different implications for both cancer control strategies and policy evaluation. First, these geospatial methods of disaggregating patient-location data to approximate a more precise location based on underlying population density allow for more granular, discriminative mapping of higher-risk areas while also maintaining patient confidentiality and data stability. There are marked variations in neighborhood environments, including racial and socioeconomic segregation, transportation resources, or carcinogen exposures, that occur in areas smaller than a county or ZIP code. Such methods could improve cancer control measurement, evaluation, and planning for departments of health, as well as informing targeted policies to address place-based drivers of adverse cancer outcomes. Given the very little overlap of incidence or late-stage hotspots, more cancer-site-specific strategies should be employed to identify unique geographic locations at higher risk of developing new cancers or cancer-site-specific factors at risk of late-stage diagnoses.

These results should be considered in the context of methodological limitations. First, the cancer registry data, although maintained with standardized variable definitions and reporting processes, are susceptible to coding errors that are often not immediately appreciated. Perhaps more relevant to the present study is that the ISCR lacks patients’ residential history which has significance, especially for incidence hotspots. Therefore, we are only able to map the locations of where patients lived at the time of diagnosis, without knowing the duration of residence or prior residencies. Since this methodology is an ongoing area of research and development, the current applications prevent determining the specific age-adjusted incidence rates or precise proportion of patients with late-stage cancer in each of these hotspots. However, our hotspots are similar in location (although more granular) compared to previously published cluster detection in Indiana, and we were able to estimate the proportion of hotspots’ incidence and late-stage cancer. Additionally, we are unable to discern additional geospatial attributes of these hotspots as many demographic or environmental data are available only at larger aggregated areas, such as census tracts, ZIP codes, or counties. As such, these methods provide more geographic granularity in describing the location of hotspots, but are unable to further describe characteristics of the populations within hotspots. Finally, our findings may be state-specific and may not be generalizable outside of Indiana. The increasing recognition of both spatial and aspatial drivers of care will require more population-specific measurements and evaluations.

## Conclusion

In this study of 133,773 patients in Indiana diagnosed with breast, colorectal, or lung cancer between 2010 and 2019, we found considerable spatial deviation of incidence and late-stage hotspots both within and between sites of disease. Within each cancer site, there was little overlap between incidence and late-stage hotspots, suggesting different cancer control measures for prevention and early detection within cancer sites. Incidence hotspots for breast and colorectal cancer were most likely to overlap, raising the potential for geographically collocating strategies for prevention of breast and colorectal cancer development. These results demonstrate the critical importance of including spatial data while identifying disparities in cancer prevention, cancer control, and timely cancer care delivery.

## Supplementary Information

Below is the link to the electronic supplementary material.

Supplementary file1 (DOCX 150 KB)

Supplementary file2 (DOCX 15 KB)

## Data Availability

This study used data available under review and a data use agreement with the Indiana State Cancer Registry. Requests to access data will be directed to the Registry at (https://www.in.gov/health/cdpc/cancer/cancer-registry/).
