# Supplementary material for: Geospatial heterogeneity of hotspots for incidence and late-stage diagnosis of breast, colorectal, and lung cancer
Source: Cancer Causes Control. 2026 May 26;37(6):90. doi: 10.1007/s10552-026-02170-z (PMC13212680; doi:10.1007/s10552-026-02170-z)

**Online Resource 1.** Age range groups for breast, colorectal and lung cancer hotspot analysis

|  | **Breast** | **Colorectal** | **Lung** |
| --- | --- | --- | --- |
| Group 1 | 18-49 | 18-49 | 18-49 |
| Group 2 | 50-74 | 50-84 | 50-79 |
| Group 3 | >75 | >85 | >80 |

**Online Resource 2.** Schematic of how the Restricted and Controlled Monte Carlo (RCMC) coupled with Unrestricted and Controlled Monte Carlo (UCMC) simulation process is performed to determine geospatial hotspots of outcomes.


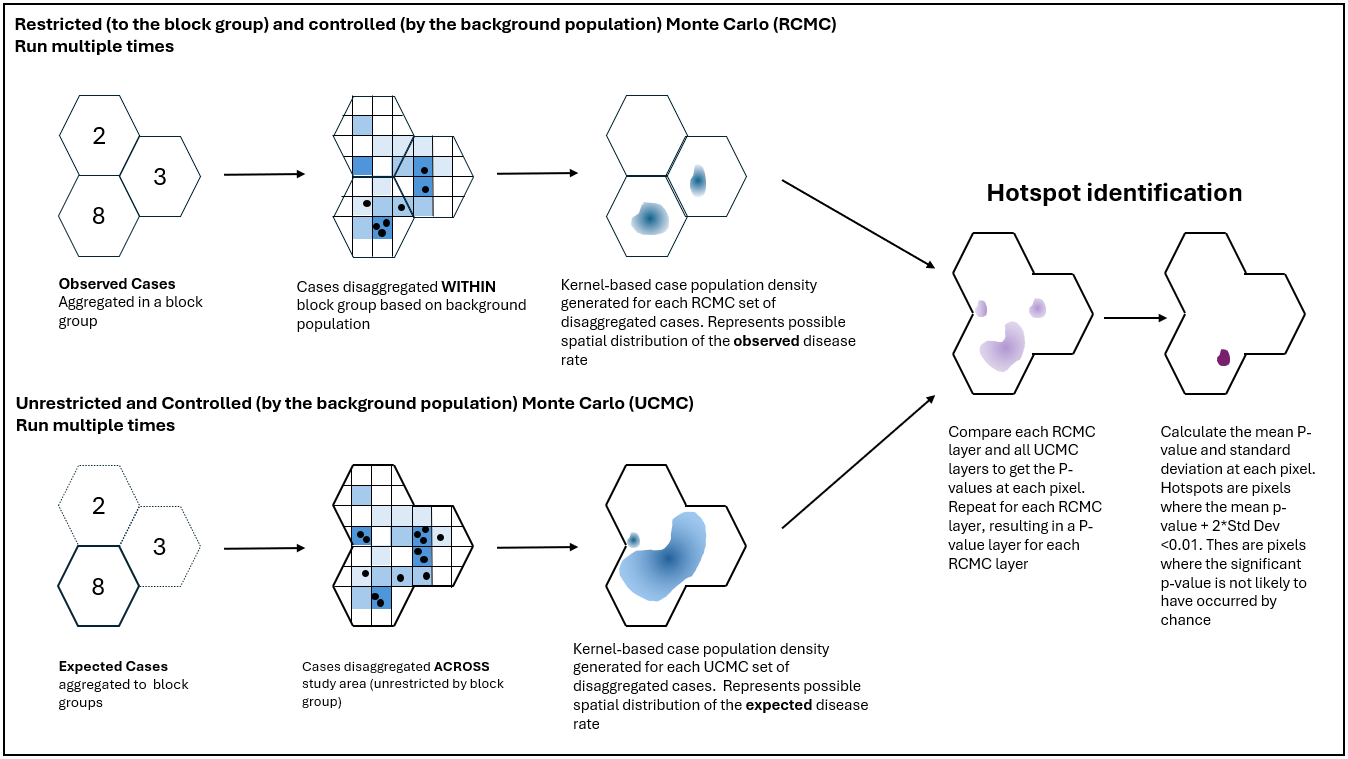

Supplement: Supplementary file 1 — Supplementary file1 (DOCX 150 KB) [file 10552_2026_2170_MOESM1_ESM.docx]
