# Supplementary material for: Geospatial heterogeneity of hotspots for incidence and late-stage diagnosis of breast, colorectal, and lung cancer
Source: Cancer Causes Control. 2026 May 26;37(6):90. doi: 10.1007/s10552-026-02170-z (PMC13212680; doi:10.1007/s10552-026-02170-z)
Supplement: Supplementary file 2 — Supplementary file2 (DOCX 15 KB) [file 10552_2026_2170_MOESM2_ESM.docx]

**Supplemental Table 2.** Proportion of the total state area for each hotspot and the proportion of hotspot overlap within and between cancer sites.

|  |  |  |  |  |  |  |  |  |  |  |  |
| --- | --- | --- | --- | --- | --- | --- | --- | --- | --- | --- | --- |
|  | **Primary Hotspots**  (% of total state area) | | | | | **Overlap of Hotspots Between Cancer Sites^†^**  (proportion of hotspots that overlap) | | | | | |
|  | **Breast** | | **Colorectal** | | **Lung** | **Breast - Colorectal** | **Breast-Lung** | | **Colorectal-Lung** | | **Breast-Colorectal-Lung** |
| **Incidence Hotspots** | **2.9%** | | **2.2%** | | **0.4%** | 15.8% | 0.6% | | 1.2% | | 0.2% |
| **Late-Stage Hotspots** | **7.3%** | | **4.4%** | | **6.0%** | 6.0% | 15.3% | | 12.1% | | 0.03% |
| **Overlap of Hotspots***  (Proportion of hotspots that overlap) | 4.7% | | 7.9% | | 8.0% |  |  | |  | |  |
| * Denominator is the area of the incidence hotspots  **^†^** Denominator is the area of the first cancer mentioned in the column heading | | | | | | | | | | | |
|  |  |  | |  | |  | |  | |  |  |
